# MicroRNA miR-223 modulates NLRP3 and Keap1, mitigating lipopolysaccharide-induced inflammation and oxidative stress in bovine mammary epithelial cells and murine mammary glands

**DOI:** 10.1186/s13567-023-01206-5

**Published:** 2023-09-14

**Authors:** Man Zhou, Herman W. Barkema, Jian Gao, Jingyue Yang, Yue Wang, John P. Kastelic, Sohrab Khan, Gang Liu, Bo Han

**Affiliations:** 1 Department of Clinical Veterinary Medicine, College of Veterinary Medicine, China Agricultural University, Beijing, 100193 China; 2 Faculty of Veterinary Medicine, University of Calgary, Calgary, AB T2N 4N1 Canada

**Keywords:** Bovine mastitis, Bta-miR-223, NLRP3 inflammasome, oxidative stress, Keap1/ Nrf2 signaling

## Abstract

Bovine mastitis, the most prevalent and costly disease in dairy cows worldwide, decreases milk quality and quantity, and increases cow culling. However, involvement of microRNAs (miRNAs) in mastitis is not well characterized. The objective was to determine the role of microRNA-223 (miR-223) in regulation of the nucleotide-binding oligomerization domain-like receptor containing pyrin domain 3 (NLRP3) inflammasome and kelch like ECH-associated protein 1 (Keap1)/nuclear factor erythroid 2-related factor 2 (Nrf2) oxidative stress pathway in mastitis models induced by lipopolysaccharide (LPS) treatment of immortalized bovine mammary epithelial cells (bMECs) and murine mammary glands. In bMECs cultured in vitro, LPS-induced inflammation downregulated bta-miR-223; the latter interacted directly with the 3’ untranslated region (3’ UTR) of NLRP3 and Keap1. Overexpression of bta-miR-223 in bMECs decreased LPS and Adenosine 5’-triphosphate (ATP)-induced NLRP3 and its mediation of caspase 1 and IL-1β, and inhibited LPS-induced Keap1 and Nrf2 mediated oxidative stress, whereas inhibition of bta-miR-223 had opposite effects. In an in vivo murine model of LPS-induced mastitis, increased miR-223 mitigated pathology in the murine mammary gland, whereas decreased miR-223 increased inflammatory changes and oxidative stress. In conclusion, bta-miR-223 mitigated inflammation and oxidative injury by downregulating the NLRP3 inflammasome and Keap1/Nrf2 signaling pathway. This study implicated bta-miR-223 in regulation of inflammatory responses, with potential as a novel target for treating bovine mastitis and other diseases.

**Supplementary Information:**

The online version contains supplementary material available at 10.1186/s13567-023-01206-5.

## Introduction

Bovine mastitis causes huge economic losses and reduces animal welfare worldwide [1]. Most intramammary infections occur when bacteria enter the mammary gland through the teat canal. As mammary epithelial cells are ubiquitously distributed throughout the mammary gland, they are typically infected due to direct contact with the pathogen; therefore, they act as the first line of physical and immunological defenses [2]. When *Escherichia coli* (*E. coli*) invades the mammary gland, lipopolysaccharide (LPS) or endotoxin, derived from Gram-negative bacteria, is continuously released into the teat lumen. Paradoxically, antimicrobial therapy increases endotoxin release and exacerbates inflammation [3]. Therefore, elucidating pathogenic features and therapeutic targets of LPS are critical to control *E. coli*-induced intramammary infections.

Inflammation and oxidative stress are important host responses of the innate immune system against infection and injury, mediated by multiple pathways and factors [2, 4]. There are many responses to inflammation induced by LPS, including multiple cell-signaling mechanisms, including DNA damage [5], reactive oxygen species (ROS) formation [6–8], plus changes in cytokine or chemokine concentrations [5, 9–11] and cellular redox balance [8, 12]. The nucleotide-binding oligomerization domain-like receptor containing pyrin domain 3 (NLRP3) inflammasome, including NLRP3, apoptosis-associated speck-like protein (ASC) containing a CARD (PYCARD) and caspase 1 as core components in the inflammasome pathway that activates pro-IL-1β and pro-IL-18 to IL-1β and IL-18 [13], has been implicated as a pathogenic mechanism in a wide variety of inflammatory conditions and considered a target for therapeutic intervention [13–15]. Oxidative stress is another aspect of mastitis-related inflammation [4, 8, 16]. The kelch like ECH-associated protein 1 (Keap1)/ nuclear factor erythroid 2-related factor 2 (Nrf2) system may concomitantly regulate both oxidative stress and anti-inflammatory responses in various models [6, 7, 17, 18]. Additionally, ROS may participate in NLRP3 inflammasome activation and the Keap1/Nrf2 oxidative stress pathway [6, 7, 19]. Given these overlaps, it may be possible to identify a common molecular target for mitigating mammary injury.

MicroRNAs (miRNAs) are small non-coding RNAs that post-transcriptionally regulate gene expression by interacting with the 3'-untranslated region (3’-UTR) of target mRNA sequences to prevent translation or promote mRNA degradation [20]. Recently, miRNA was closely linked to regulation of inflammation and oxidative stress. There is limited knowledge of miRNA expression and the role of these molecules during mastitis; however, such data would provide evidence of potential roles in occurrence and development of bovine mammary disease, plus expand knowledge of diversity of *Bos taurus* miRNA [21, 22]. Recent studies revealed important regulatory roles of miRNAs during bovine mammary gland infections [21, 22]. For instance, > 50 miRNAs were identified; miR-21, miR-23a, miR-24 and miR-143 were more abundantly expressed in the mammary gland than other tissues, and miR-21 stimulated cell growth [22]. In addition, 15 miRNAs had upregulated and 7 miRNAs downregulated expression in cultured bovine mammary epithelial cells challenged with *Streptococcus uberis* [23]. Furthermore, in bovine monocytes challenged with LPS or *Staphylococcus aureus* enterotoxin B, 5 miRNAs (miR-9, miR-125, miR-155, miR-146a and miR-223) were involved in the inflammatory response [24].

Identifying important miRNAs and their interactions with target genes should provide insights regarding their regulatory functions. Hundreds of miRNA genes have been identified in animals, with miR-223 implicated in antimicrobial immunity and inflammation [25] as well as in innate immunity, inflammation, and cancer [21, 26–28]. Furthermore, miR-223 regulated a wide range of target genes and simultaneously modulated multiple pathways in various models [14, 29, 30]. For example, hepatic neutrophils were key contributors to reversing inflammation, and proinflammatory macrophages were key regulators of hepatic injury and fibrosis activated by persistent inflammation. The former might induce the latter into a restorative inflammatory phenotype via miR-223 [29]. In a diabetes mellitus model, miR-223 regulated the Keap1/Nrf2 pathway to affect oxidative stress in a hepatocarcinoma cell line [30]. However, the role of miR-223 during mastitis is largely unknown. Therefore, our objective was to determine the role of miR-223 in regulation of the NLRP3 inflammasome and Keap1/Nrf2 oxidative stress pathway in mastitis models involving LPS treatment of immortalized bovine mammary epithelial cells (bMECs) and murine mammary glands.

## Materials and methods

### Statement of ethics

This study received approval for research ethics from the Ethical Committee of the College of Veterinary Medicine, China Agricultural University (CAU), Beijing, China (Protocol SYXK, 2016-0008). Furthermore, it was conducted according to standard ethical guidelines enforced at CAU.

### Cell culture, treatment and transfection

Immortalized bovine mammary epithelial cells (bMECs) retain features and functions of primary cells, with an absence of senescence [31, 32]. The bMECs were cultured in specialty media before treatment and cultivated stably with routine subculturing, as described [32, 33]. Before experiments, bMECs were cultured in Dulbecco’s modified Eagle’s medium (DMEM) for 1 week without treatment. LPS (L2880, LPS from *E. coli* O55:B5, Sigma-Aldrich, St. Louis, MO, USA) and/or ATP (SL1260, Coolaber Technology Co., Ltd, Beijing, China) was used as an inflammatory challenge. MCC950 (S7809, Selleck Chemicals, Shanghai, China), an NLRP3 inflammasome inhibitor, was used to attenuate cellular NLRP3 inflammasome activity.

The bMECs were seeded at a density of 1 × 10^5^ cells in a 6-well plate for subsequent experiments. Transfections were performed with short interfering RNA (siRNA)-Mate Reagent (GenePharma Co. Ltd, Suzhou, China), according to manufacturer’s instructions. To establish bta-miR-223 overexpression or inhibition, bMECs were transfected with bta-miR-223 mimics (sense: 5’-UGUCAGUUUGUCAAAUACCCCA-3’; antisense: 5’-GGGUAUUUGACAAACUGACAUU-3’) or inhibitor (sense: 5’-UGGGGUAUUUGACAAACUGACA-3’) and the corresponding control for 24 h before LPS treatment. The miRNA mimics, inhibitor and NC were synthesized by GenePharma Co. Ltd (Suzhou, China). Bta-miR-223 mimics or inhibitor and their matched NC were added to 6-well culture plates and cells subjected to assays or extraction of RNA or protein.

### Murine mastitis model and treatment

Female Balb/C mice (*n* = 36, pregnant 21 days) were housed in the laboratory animal room of the Experimental Animal Center of China, with ad libitum access to standard pellet feed and water. These mice were randomly allocated into 6 groups: 1) saline and agomir NC injection (agomir NC group); 2) LPS infection and agomir NC injection (LPS group); 3) LPS infection and overexpression of miR-223 (agomiR-223) injection (agomiR-223 group); 4) saline and antagomir NC injection (antagomir NC group); 5) LPS infection and antagomir NC injection (LPS group); and 6) LPS infection and inhibition of miR-223 injection (antagomiR-223 group). The miRNAs agomir and antagomir were purchased from GenePharma Co. Ltd. A model of LPS-induced murine mastitis (10 μg/100 μL of LPS injected into each nipple) was performed as described in our previous studies [34]. For in vivo transfection, 48 and 24 h before mammary gland injection, mice received intraperitoneal injections of 375 μL agomiR-223 (final concentration 20 μmol/L) or 750 μL antagomiR-223 (final concentration 20 μmol/L). At 12 h after LPS stimulation, all mice were euthanized with an overdose of sodium pentobarbital and mammary tissues immediately excised.

### Cell viability assay

To investigate effects of LPS on bMECs and screen appropriate concentrations and exposure times for LPS, cell viability was determined using a Cell Counting Kit-8 (CCK-8; Beyotime Biotechnology, Shanghai, China) reduction assay. The CCK-8 was used to enumerate living cells by combining WST-8 (2-(2-methoxy-4-nitrophenyl)-3-(4-nitrophenyl)-5-(2,4-disulfophenyl)-2H-tetrazolium) and 1-methoxy PMS (1-methoxy-phenazine methosulfat). Briefly, the bMECs were seeded into 96-well plates. After cells reached 80% confluence, they were treated with LPS (0, 0.1, 1, 10, 50, or 100 μg/mL) for 6, 12 or 24 h, then CCK-8 reagent (10 μL) was added to each well and incubated for 2 h. Absorbance was recorded on a microplate reader at a wavelength of 450 nm.

### Lactate dehydrogenase (LDH) release assay

To evaluate LPS cytotoxicity, LDH release was assessed with an LDH Assay Kit (Nanjing Jiancheng Bioengineering Institute, Nanjing, China). LDH, a glycolytic enzyme in the cytoplasm, is released into the supernatant medium following loss of membrane integrity. Briefly, bMECs were plated in 96-well plate with 10^5^/well. After LPS stimulation (0, 0.1, 1, 10, 50, or 100 μg/mL for 6, 12 or 24 h), spent medium from bMECs was collected and centrifuged (2500 × *g* for 10 min) and LDH concentrations were determined at 450 nm with a microplate reader, as described above.

### Enzyme-Linked Immunosorbent Assay (ELISA)

Quantitation of cytokines is of great interest in clinical trials due to their roles in inflammatory disease states and maintaining homeostasis. Immunoassay-based techniques are commonly used to measure cytokine concentrations. Firstly, bMECs were seeded in 6-well plates overnight. At 3, 6, 9, 12, and 15 h after LPS-stimulation, concentrations of inflammatory cytokines, IL-1β, IL-18, TNF-α, and IL-6 in the supernatants were determined with ELISA kits (Mlbio, Shanghai, China), according to the manufacturer’s protocol.

### Western blotting (WB) assay

After the indicated treatments, bMECs and mammary gland tissues were lysed with radioimmunoprecipitation assay (RIPA) lysis buffer (Beyotime Biotechnology Co. Ltd, Shanghai, China) and protein concentrations determined (Bicinchoninic acid (BCA) Protein Assay Kit, CWBIO, Beijing, China). Then, WB was performed using standard protocols. The following primary antibodies were used: nuclear factor-κ-gene binding protein (NF-κB), p65 (Proteintech #10745-1-AP, 1:1000); Phospho-NF-κB p65 (Ser536) (Bioss #bs-0982R, 1:1000); inhibitor of NF-κB (IκB) (Proteintech #10268-1-AP, 1:1000); Phospho-IκB (Ser36) (Bioss #bs-18129R, 1:1000); NLRP3 (Bovine) (Proteintech #19771-1-AP, 1:500); NLRP3 (Mouse) (Abcam #ab263899, 1:1000); caspase 1 (Proteintech #22915-1-AP, 1:1000); IL-1β (Wanleibio #WL00891, 1:500); Keap1 (Proteintech #10503-1-AP, 1:1000); Nrf2 (S40) (Abmart #T56573, 1:500); heme oxygenase-1 (HO-1) (Abcam #ab13243, 1:1000); superoxide dismutase 1 (SOD1) (Wanleibio #WL01846, 1:1000); toll-like receptor 4 (TLR4) (Proteintech #19811-1-AP, 1:1000); beta actin (Proteintech #20536-1-AP, 1:2000) and beta tubulin (Proteintech #10094-1-AP, 1:2000). Secondary horseradish peroxidase (HRP)-conjugated anti-rabbit (CWBIO #CW0103, 1:5000) or anti-mouse (CWBIO #CW0102, 1:5000) antibodies were applied and allowed to bind primary antibodies, and the immune reaction was then detected with an enhanced chemiluminescence system (ECL; Beyotime Biotechnology Co. Ltd, Shanghai, China).

### Measurement of intracellular ROS

Cellular ROS promotes oxidation of DCFH to yield the fluorescent product, 2’,7’-dichlorofluorescein diacetate (DCFH-DA). Concentrations of ROS were measured via a Reactive Oxygen Species Assay Kit (Beyotime Biotechnology Co. Ltd). bMECs were pretreated with LPS (LPS group) or without LPS (Control group), and then suspended in 10 μmol/L DCFH-DA for 30 min away from light at 37 °C. bMECs treated without DCFH-DA was the negative control group, whereas bMECs treated with Rosup (a ROS activator) for 20 min at 37 °C were the positive control group. After washing with phosphate buffered solution (PBS), the mean fluorescence intensity of each group was assessed with flow cytometry (Becton, Dickinson, San Jose, CA, USA).

### Dual-luciferase reporter assay

The dual-luciferase reporter assay has been used widely to validate animal miRNA targets. Wild type (WT) and mutant type (MUT) sequences of NLRP3 3’-UTR and Keap1 3’-UTR containing the bta-miR-223 binding site were amplified and cloned into the vectors psicheck2 (Promega, Madison, WI, USA) and pmirGLO (Promega) downstream of the firefly luciferase gene, respectively. Bta-miR-223 mimic and negative control (NC) oligonucleotides were synthesized (GenePharma Co. Ltd). HEK 293 T cells (1 × 10^5^) were seeded into 12-well dishes overnight. Then, bta-miR-223 mimic or NC with WT or MUT luciferase reporter plasmid were co-transfected into 293 T cells using Lipofectamine 3000 (Invitrogen Life Technologies, Carlsbad, CA, USA) for 24 h. Luciferase activity from the collected cell lysate was quantified using a Dual-Luciferase Reporter Assay System (Promega, Madison, WI, USA) and microplate reader (BioTek Instruments Inc., Winooski, VT, USA). Relative luciferase activity was defined as the ratio between renilla luciferase activity and firefly luciferase activity.

### Fluorescence in situ hybridization (FISH)

Cells or tissue samples were fixed with 4% paraformaldehyde (Servicebio, Wuhan, China) for 12 h, dehydrated, embedded in paraffin, and sectioned. Slides were digested with Proteinase K (Beyotime Biotechnology Co. Ltd, Shanghai, China) for 30 min, pre-hybridized at 37 °C for 1 h and hybridized overnight with probes (5’-Cy3-UGGGGUAUUUGACAAACUGACA-3’; Rochen Pharma Co. Ltd, Shanghai, China). Cells and tissue were counterstained with 4,6-Diamidino-2-phenylindole (DAPI) (Solarbio Biotechnology Co. Ltd., Beijing, China) for 8 min and visualized by fluorescence microscopy using the Cy3 channel.

### Quantitative real-time PCR assay (qRT-PCR)

Total RNA was extracted from transfected cells with 1 mL TransZol Up (TransGen Biotech Co. Ltd., Beijing, China), and the RNA quality and concentration were assessed with a NanoDrop 2000 Spectrophotometer (Thermo Fisher Scientific, Waltham, MA, USA). Quantification of miRNA, generation of cDNA and detection of real time RT-PCR were done with Hairpin-it^™^ microRNA and U6 snRNA Normalization RT-PCR SYBR Green Quantitation kit (GenePharma Co. Ltd, Suzhou, China) containing a stem-loop-like RT primer and PCR primers specific for various miRNAs or the U6 RNA endogenous control. Briefly, expression of miR-223 in LPS-induced samples relative to that in normal samples was determined using the 2^−ΔΔCT^ method. All primer sequences are listed in Table 1.

**Table 1:** **miRNA primer sets for qRT-PCR**

Species	Name	Primer sequence (5ʹ–3ʹ)
Bovine	Bta-miR-223	F: GTTGCTCCTGTCAGTTTGTCAAA
		R: TATGGTTGTTCACGACTCCTTCAC
	U6	F: ATGCTTGCTTTAACAGCACATA
		R: CATCCTTGTACAGGGACCATG

### Histology and immunohistochemistry

Mammary gland tissues were excised immediately after euthanasia and fixed overnight in 4% paraformaldehyde (Servicebio, Wuhan, China). Tissues were paraffin-embedded and sectioned (5 μm), with sections dewaxed in xylene and rehydrated in an alcohol series, prior to staining with hematoxylin and eosin (HE; Beyotime Biotechnology Co. Ltd, Shanghai, China). Histological scoring was performed, without knowledge of treatment group, to grade tissue necrosis, dislodged epithelial cells, polymorphonuclear neutrophilic granulocyte inflammation, and lymphocytic infiltration, as described [35].

For immunohistochemistry (IHC), sections were deparaffinized and rehydrated. Sodium citrate buffer (pH = 6.0) was used for antigen retrieval. Sections were washed 3 times in PBS, incubated in PBS with 0.5% Triton X-100 (Solarbio Biotechnology Co. Ltd., Beijing, China) for 15 min and then incubated in block solution. Then sections were immunostained with primary antibodies NLRP3 (Servicebio #GB11300, 1:1000) or Keap1 (Servicebio #GB11847, 1:2000) at 4 °C overnight. Thereafter, sections were incubated with HRP-conjugated secondary antibodies (Servicebio #GB23303, 1:200) and stained with 3,3’-diaminobenzidine (DAB) (Servicebio #G1211).

### Statistical analyses

All experiments were repeated at least 3 times. One-way ANOVA was used to compare, among groups, relative luciferase activities, cell viability, LDH activities, intracellular ROS concentrations, histological scores, effects of LPS and/or siRNA on miRNA expression and protein expression levels, in bMECs and murine mammary tissue. Data were presented as mean ± standard deviation (SD). SPSS 26.0 (SPSS Inc., Chicago, IL, USA) was used for statistical analyses, with *p < 0.05 and **p < 0.01 considered significantly and extremely significantly different, respectively. Histograms were produced with Graphpad Prism 8.0 (GraphPad Software, Inc., San Diego, CA, USA).

## Results

### LPS challenge increased inflammation and oxidative stress in bMECs

**Figure 1:**
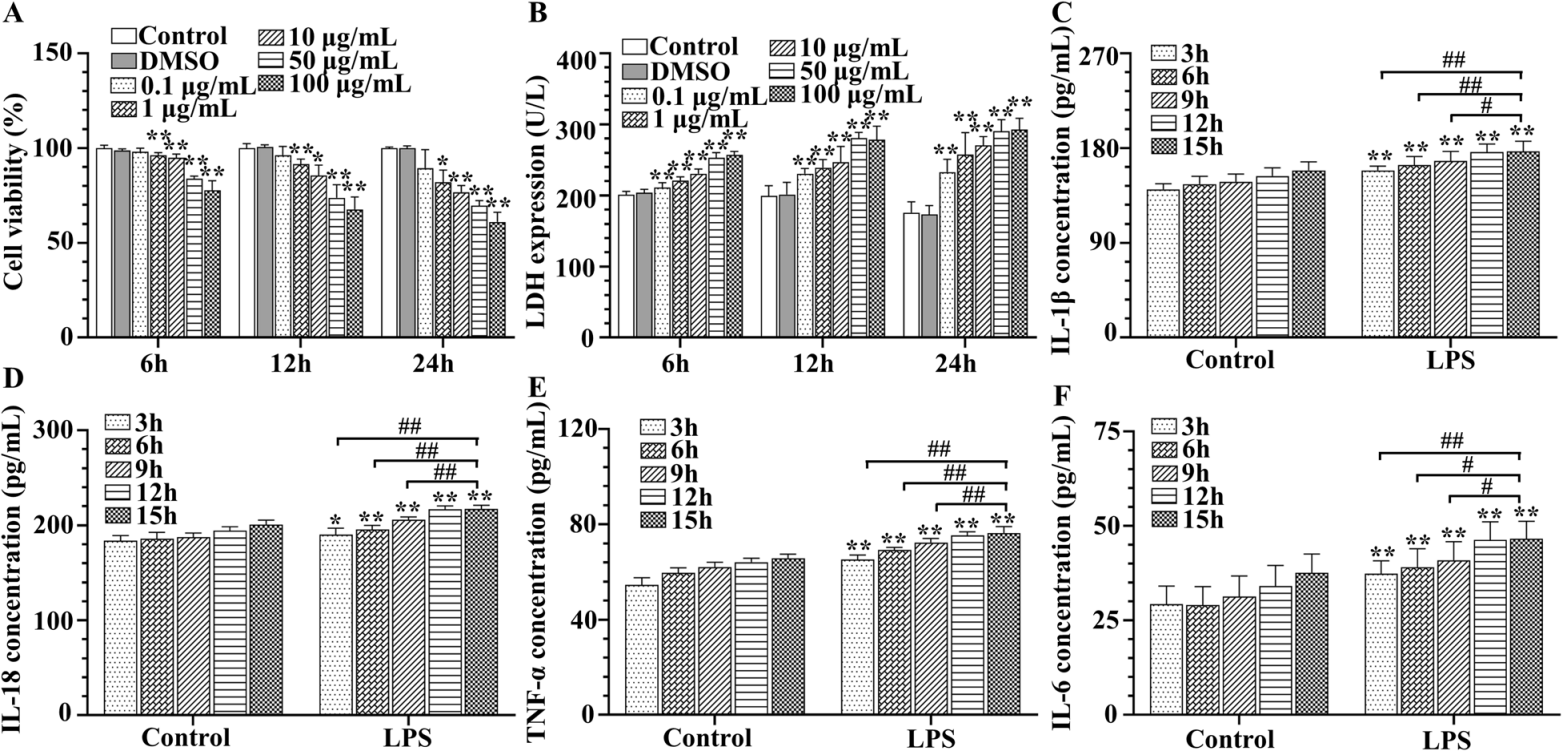
**Effects of doses and timing of LPS on immortalized bMECs. A** Cell viability was determined with a CCK-8 assay. **B** LDH release was detected by LDH Assay Kit. **C**–**F** Concentrations of IL-1β **(C)**, IL-18 **(D)**, TNF-α **(E)** and IL-6 **(F)** in the supernatant was quantified by ELISA. *p < 0.05 and **p < 0.01, differences compared to control group. #*p* < 0.05 and ##*p* < 0.01, differences compared to LPS-induced samples.

To assess cell viability (Figure 1A) and cytotoxic effects (Figure 1B) of LPS, bMECs were cultured with various concentrations of LPS for 6, 12 or 24 h, and cellular viability and LDH activity were measured. Compared to control or dimethyl sulfoxide (DMSO) treated groups, cell viability of bMECs incubated with LPS was not significantly altered at the concentration of 0.1 µg/mL, but was lower (*p* < 0.01) at concentrations of 50 or 100 µg/mL in all time points (Figures 1A). However, only after 24 h, cell viability following incubation with the 2 concentrations of LPS (50 or 100 μg/mL) was decreased close to 50% (*p* < 0.01) (Figure 1A). Based on cytotoxic LDH release assay, there was an obvious increase after treatment with 50 μg/mL LPS for 12 h compared to other infection groups (*p* < 0.01) (Figure 1B). In addition, LPS-induced release of LDH increased with both LPS concentration and time (*p* < 0.01) (Figure 1B).

ELISA assays of cytokine concentrations in supernatants of co-cultures indicated significant changes (Figures 1C–F) throughout repeated experiments. Treatment with 50 μg/mL LPS increased (*p* < 0.01) release of several cytokines (IL-1β, IL-18, TNF-α, and IL-6) at multiple time points (Figure 1C–F), with no significant difference between 50 and 100 μg/mL LPS in the amount of cytokines released (attributed to a plateau). Based on these outcomes, bMECs treated with 50 μg/mL LPS for 12 h were chosen for subsequent studies.

**Figure 2:**
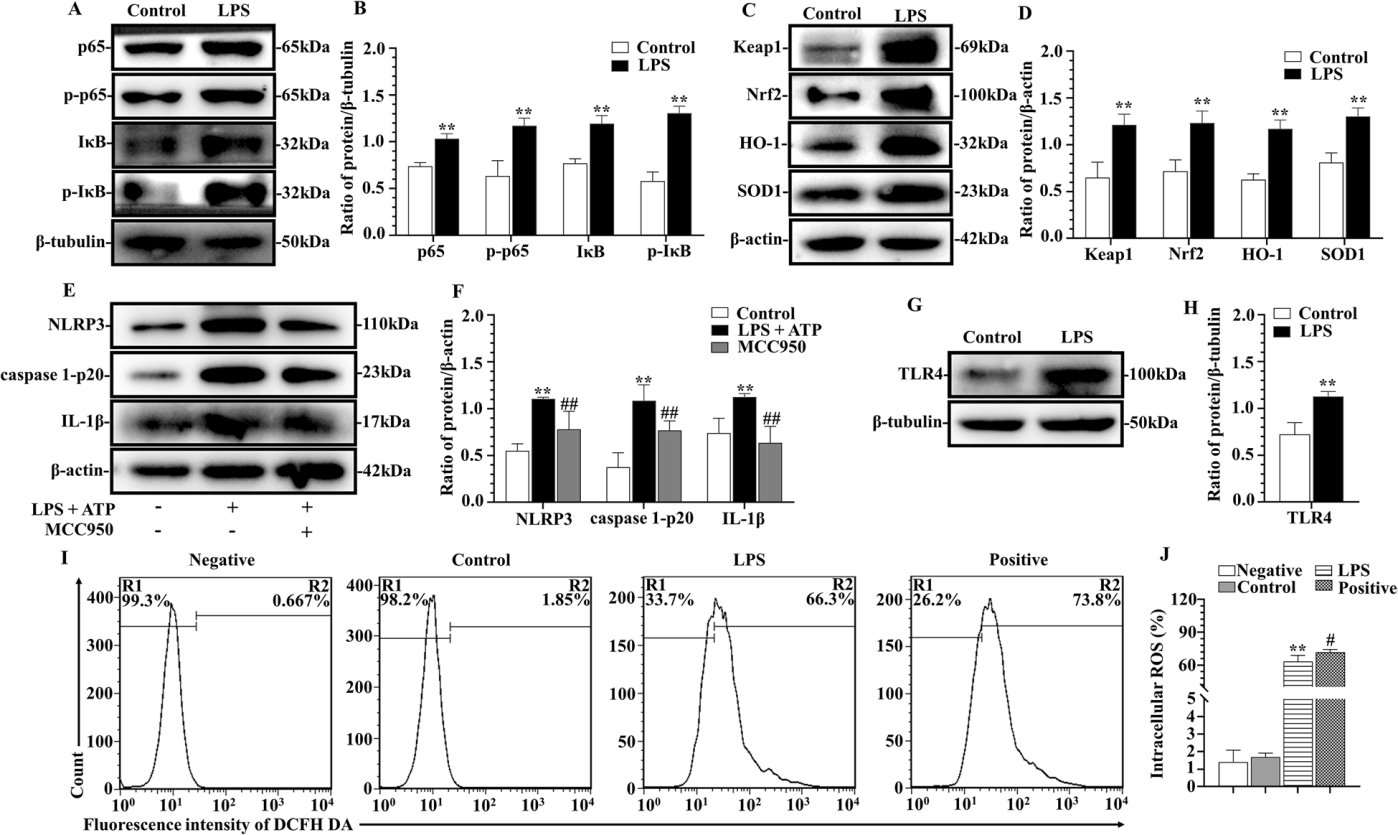
**Effects of inflammation and oxidative stress of LPS on immortalized bMECs. A**, **B** NF-κB signaling pathway activation of bMECs induced by LPS. **C**, **D** Keap1/Nrf2 signaling pathway activation of bMECs induced by LPS. **E**, **F** NLRP3 inflammation pathway activation of bMECs induced by LPS and ATP (10 mmol/L) for 1 h and/or MCC950 (10 μmol/L) for 12 h. **G**, **H** Protein expression of TLR4 of bMECs induced by LPS. **I**, **J** Intracellular ROS production of bMECs induced by LPS. **p < 0.01, differences compared to control group. #*p* < 0.05 and ##*p* < 0.01 differences compared to LPS or LPS + ATP-induced samples.

Treatment of bMECs with 50 μg/mL LPS increased inflammatory responses and oxidative stress in bMECs after 12 h (Figure 2). Compared to the control group, there was a significant increase in intracellular ROS in the LPS group within the screening concentration and incubation time (*p* < 0.01), which approached the ROS concentration in the DCFH-DA-positive group (Figures 2I, J). Furthermore, this challenge also increased activation of the NF-κB pathway (Figures 2A, B) and NLRP3 inflammasome pathway (Figures 2E, F) compared to the control. As the intracellular NLRP3 concentration is vital for assembly and activation of the NLRP3 inflammasome, we quantified inflammatory cytokines (IL-1β) and NLRP3 inflammasome components (NLRP3 and caspase 1); and levels of all proteins were upregulated in bMECs stimulated by LPS + ATP (*p* < 0.01) (Figures 2E, F), but downregulated in the LPS + ATP + MCC950 (NLRP3 inhibitor) group (*p* < 0.01) (Figures 2E, F). Regarding the NF-κB signaling pathway, TLR4, p65 and its phosphorylation (p-p65) and IκB and its phosphorylation (p-IκB) were all upregulated (*p* < 0.01) (Figures 2A, B, G and H). In addition, based on the Keap1/Nrf2 oxidative stress axis, both Keap1 and Nrf2 were upregulated and several downstream proteins, including HO-1 and SOD1, were also upregulated in bMECs (*p* < 0.01) (Figures 2C, D).

### Bta-miR-223 directly targeted NLRP3 and Keap1

**Figure 3:**
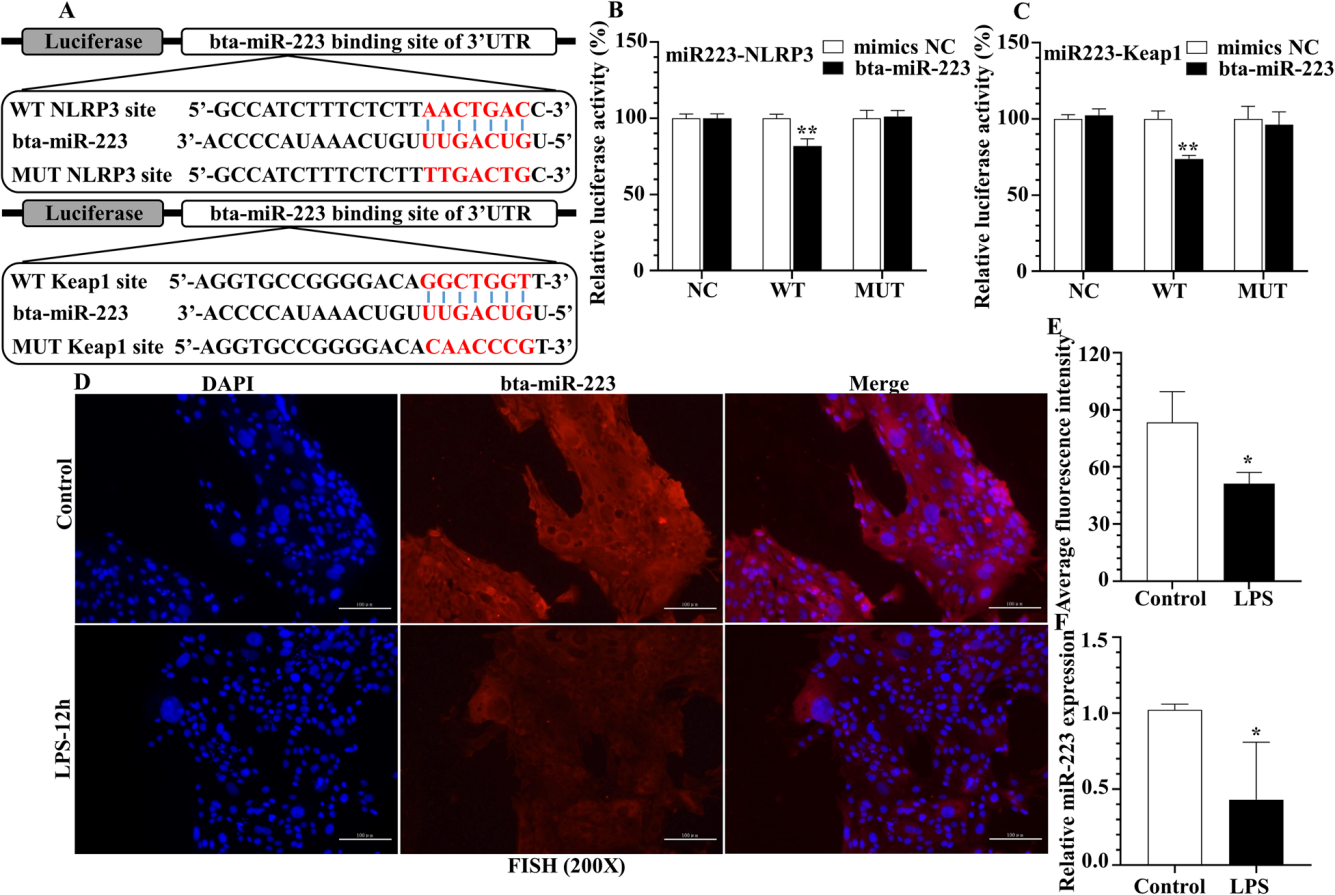
**Bta-miR-223 directly targets NLRP3 and Keap1 on immortalized bMECs, respectively. A**–**C** Homology of *Bos taurus* NLRP3 and Keap1 3’UTR and bta-miR-223. **A** The binding sites were between bta-miR-223 and the 3’UTR of NLRP3 and Keap1, respectively. **B**, **C** Data are plotted as the ratio between renilla luciferase activity and firefly luciferase activity. **D**–**F** Bta-miR-223 level in cultured bMECs challenged by LPS. **D**, **E** Intracellular levels of bta-miR-223 were quantified by FISH, scale bar, 100 μm, and **F** real-time PCR using individual SYBR Green miRNA assays. *p < 0.05 and **p < 0.01, differences compared to control group.

Computational prediction via TargetScan revealed that bta-miR-223 putatively targeted *Bos taurus* NLRP3 and Keap1 3’UTR, respectively (Figure 3A). To further verify the target relationship between miRNA and mRNA, in a dual-luciferase reporter assay, overexpression of bta-miR-223 diminished (*p* < 0.01) luciferase activity of NLRP3 and Keap1 3’UTR of the WT, respectively (Figures 3B, C). There was no difference in luciferase activity of the NC plasmid group or MUT group (*p* > 0.05) (Figures 3B, C). Therefore, we inferred that bta-miR-223 specifically bound to the *Bos taurus* NLRP3 and Keap1 3’UTR in the 293 T cell system, respectively.

Both FISH and qRT-PCR analysis of bta-miR-223 expression from bMECs were done (Figures 3D, F). After bta-miR-223 was labeled with fluorophores, its expression profile was visualized. Based on qRT-PCR, kinetics of cellular bta-miR-223 expression were examined in bMECs at 12 h after LPS stimulation (50 μg/mL); for both methods, there was a modest decrease in bta-miR-223 expression compared to the control (Figures 3E, F).

### Bta-miR-223 suppressed NLRP3/caspase 1/IL-1β and Keap1/Nrf2 pathways in bMECs

**Figure 4:**
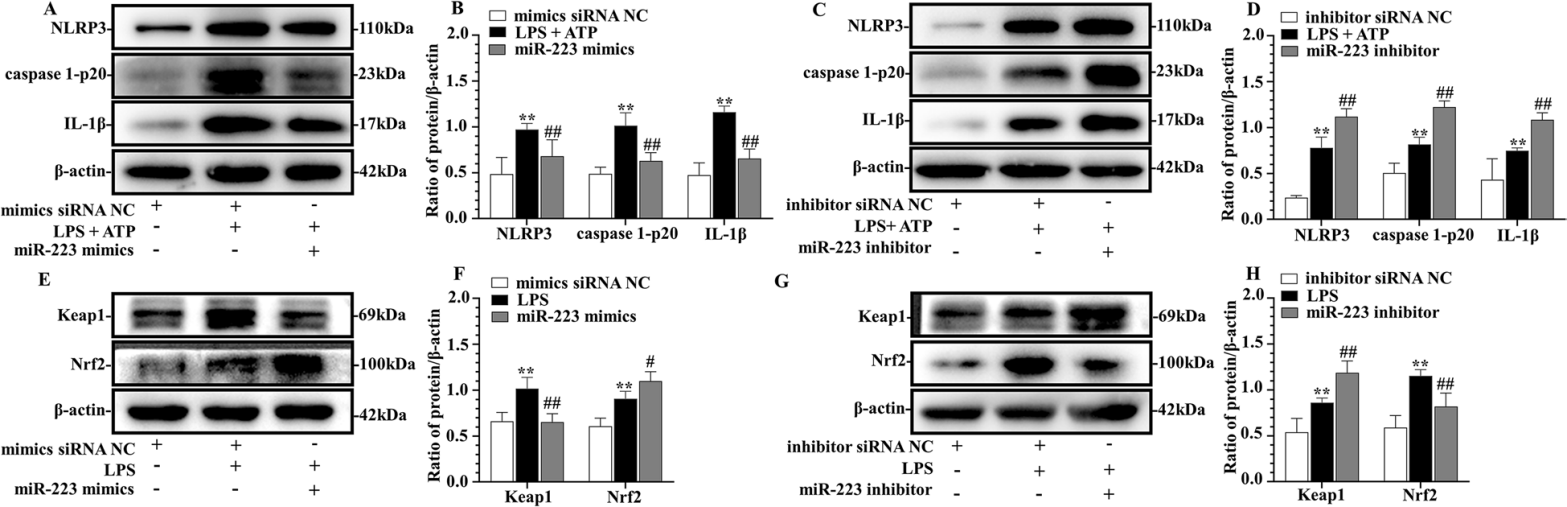
**Establishment of exogenous bta-miR-223 mimics (A, B, E, F) and bta-miR-223 inhibitor (C, D, G, H) on immortalized bMECs.** Effects of bta-miR-223 mimics **(A**, **B)** or bta-miR-223 inhibitor **(C**, **D)** on NLRP3 pathway induced by LPS + ATP. Effects of bta-miR-223 mimics **(E**, **F)** or bta-miR-223 inhibitor **(G**, **H)** on Keap1/Nrf2 pathway induced by LPS. **p < 0.01, differences compared to control group; #*p* < 0.05 and ##*p* < 0.01, differences compared to LPS or LPS + ATP-induced samples.

To further define a mechanistic role for bta-miR-223 in modulation of mammary inflammation and oxidative stress, bMECs were transfected with miR-223 mimics or inhibitors or corresponding NC (Figure 4) and 50 nmol/L transfection was chosen on the basis of a dose–response screening experiment (Additional file 1A and B). Exposure to LPS and/or ATP after promotion and inhibition of bta-miR-223 miRNA level decreased and increased activation of the NLRP3 inflammasome and Keap1/Nrf2 signaling pathway, respectively (Figure 4). Furthermore, bta-miR-223 mimics transfection reduced NLRP3, caspase 1-p20 and IL-1β concentration following LPS and ATP stimulation in comparison to siRNA NC (*p* < 0.01) (Figures 4A, B), whereas bta-miR-223 inhibitor had the opposite effect (*p* < 0.01) (Figures 4C, D). Moreover, overexpression of bta-miR-223 inhibited LPS-induced Keap1 and Nrf2 cascade (*p* < 0.05) (Figures 4E, F), whereas bta-miR-223 inhibitor increased expression level of Keap1 compared to corresponding siRNA NC (*p* < 0.01) (Figures 4G, H). Overall, these results confirmed that bta-miR-223 had a critical role in regulating NLRP3 inflammasome and Keap1-medidated oxidative stress in bMECs.

### miR-223 modulated NLRP3 and Keap1 in murine mammary gland

**Figure 5:**
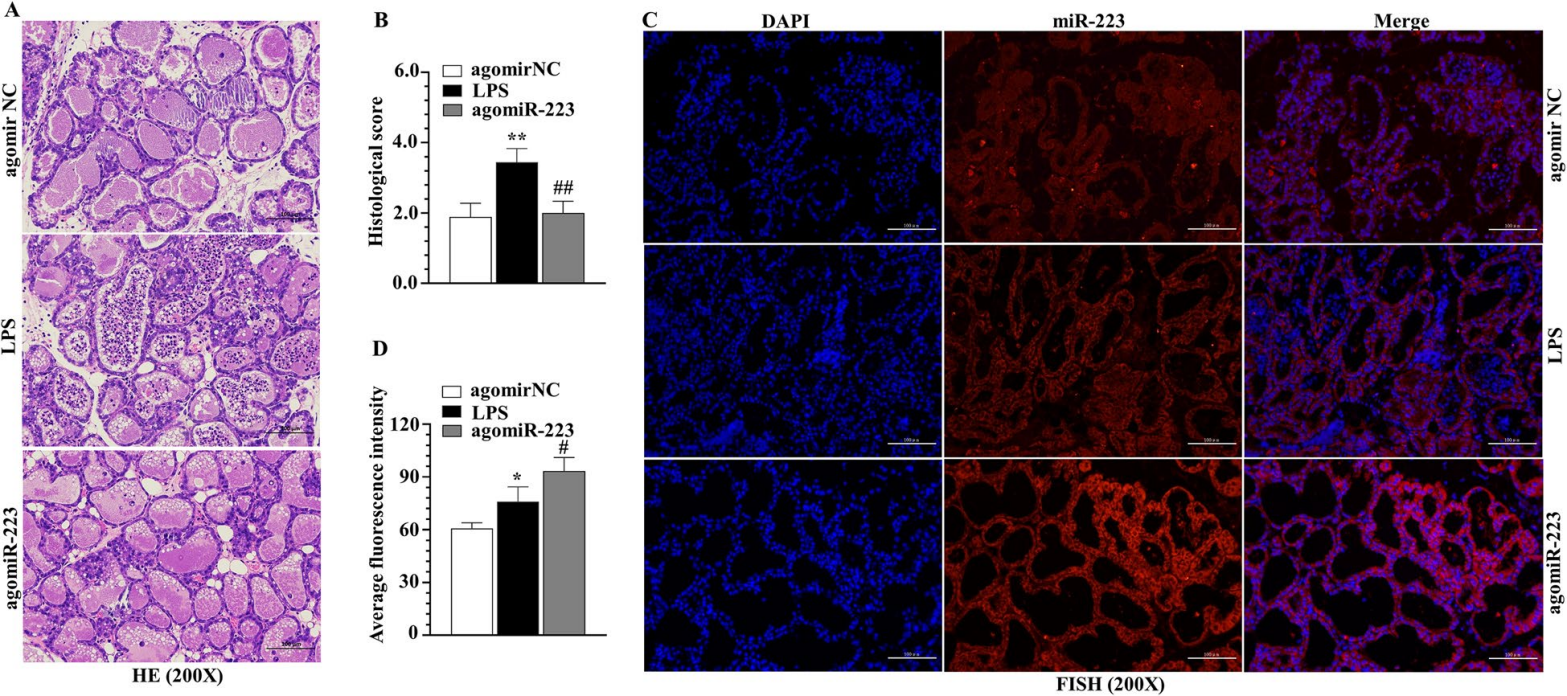
**Delivery of agomiR-223 of experimental LPS-induced mastitis. A**, **B** H&E staining and histological score of mammary gland tissue, scale bar, 100 μm. **C**, **D** miR-223 level in mammary gland tissue challenged by LPS and agomiR-223, scale bar, 100 μm. *p < 0.05 and **p < 0.01, differences compared to control group; #*p* < 0.05 and ##*p* < 0.01, differences compared to LPS-induced samples.

**Figure 6:**
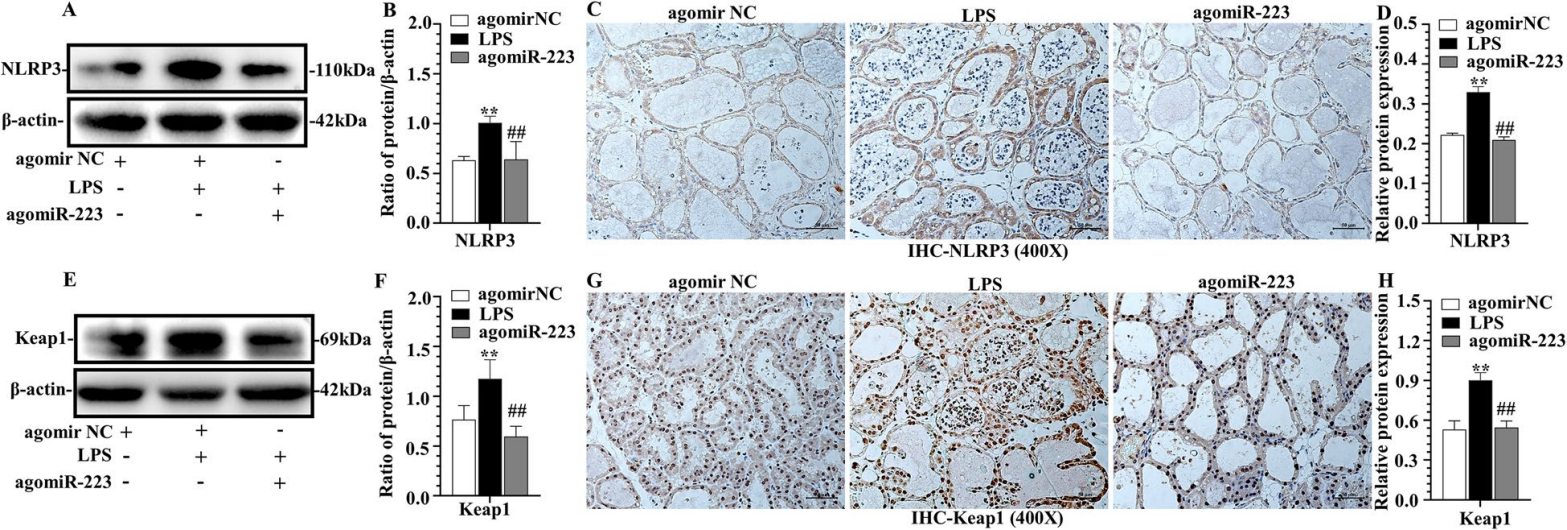
**Protein expression of murine mastitis with overexpression of miR-223.** Effect of agomiR-223 on NLRP3 **(A**–**D)** and Keap1 **(E**–**H)** expression in mammary gland tissue. NLRP3 and Keap1 were quantified by WB **(A**, **B**, **E**, **F)** and IHC **(C**, **D**, **G**, **H),** scale bar, 50 μm. **p < 0.01, differences compared to control group; ##*p* < 0.01, differences compared to LPS-induced samples.

**Figure 7:**
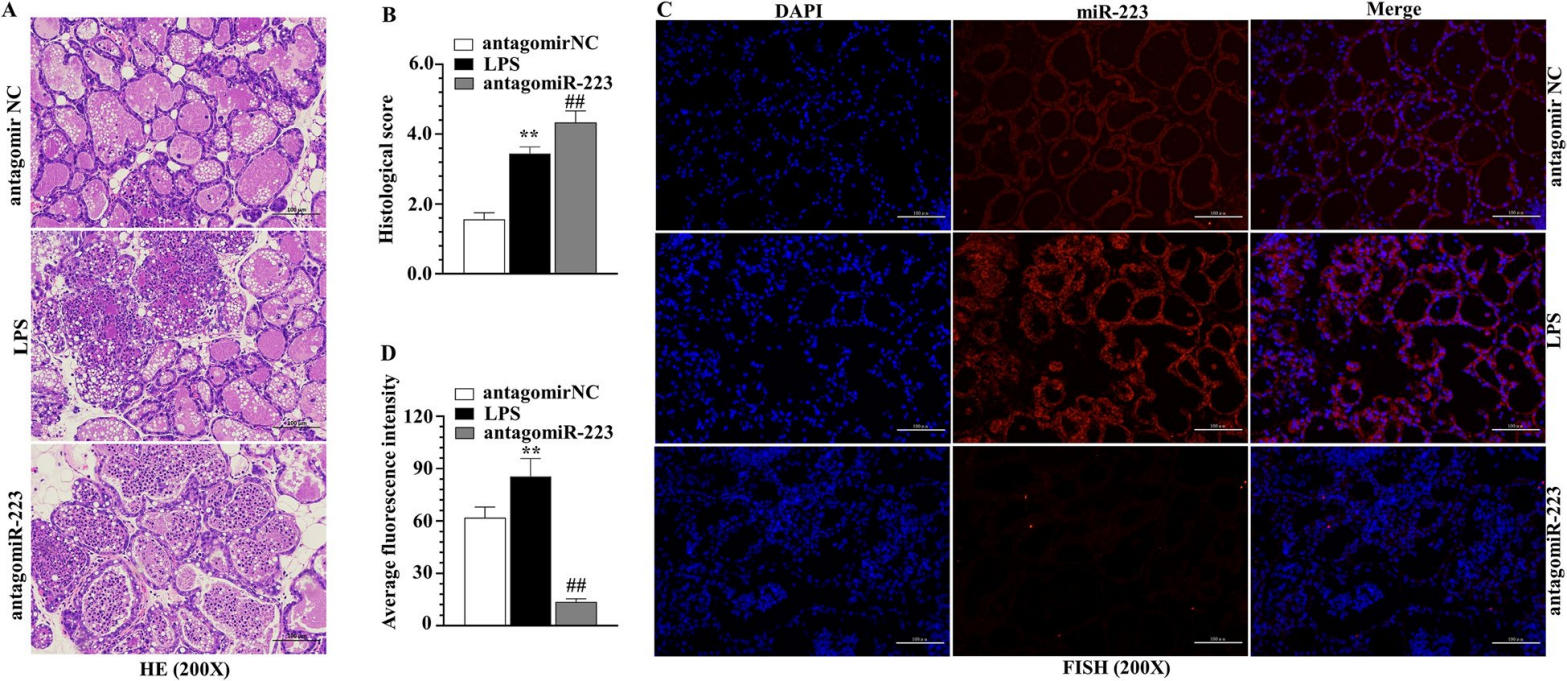
**Delivery of antagomiR-223 of experimental LPS-induced mastitis. A**, **B** H&E staining and histological score of mammary gland tissue, scale bar, 100 μm. **C**, **D** miR-223 level in mammary gland tissue challenged by LPS and antagomiR-223, scale bar, 100 μm. **p < 0.01, differences compared to control group; ##*p* < 0.01, differences compared to LPS-induced samples.

**Figure 8:**
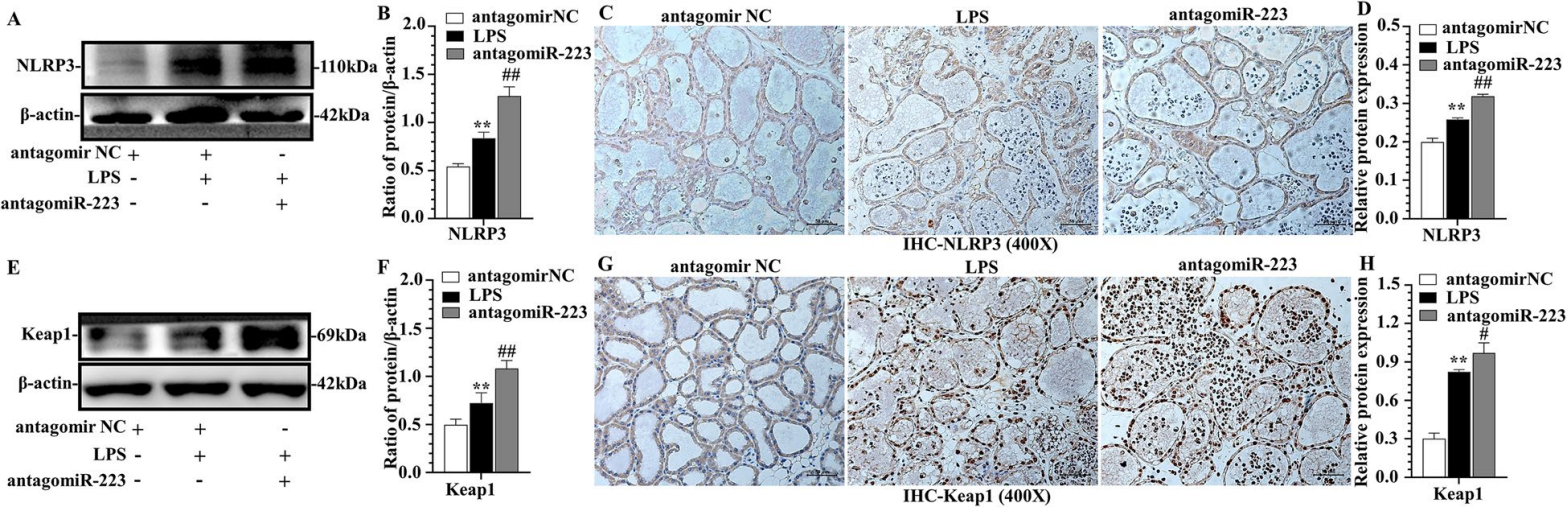
**Protein expression of murine mastitis with suppression of miR-223.** Effect of antagomiR-223 on NLRP3 **(A**–**D)** and Keap1 **(E**–**H)** expression in mammary gland tissue. NLRP3 and Keap1 were quantified by WB **(A**, **B**, **E**, **F)** and IHC **(C**, **D**, **G**, **H)**, scale bar, 50 μm. **p < 0.01, differences compared to control group; #*p* < 0.05 and ##*p* < 0.01, differences compared to LPS-induced samples.

To further investigate potential biological significance of miR-223 in vivo, pregnant mice were challenged with agomiR-223 or antagomiR-223 or corresponding NC (Figures 5, 6, 7, 8). Twice dosing within 48 h significantly increased or decreased miR-223 levels in murine mammary glands, indicting the validity of the model. In FISH assays, miR-223 levels were induced (Figures 5C, D) or depleted (Figures 7C, D) by agomir and antagomir treatments, respectively. Mammary gland tissue had severe hyperemia and edema, and milk stasis in the LPS group at 12 h post-infection compared to the siRNA NC group. Histologically, H&E staining indicated a small number of sloughed epithelial cells or infiltrated inflammatory cells in the milk ducts with overexpression of miR-223 compared to the LPS group (Figure 5A). Conversely, knockdown of miR-223 aggravated tissue damage, with massive infiltration of cells (Figure 7A), with histological scores having the same trend (Figures 5B and 7B). In addition, expression of NLRP3 and Keap1 proteins was measured by both WB (Figures 6A, B, E, F and 8A, B, E, F) and IHC (Figures 6C, D, G, H and 8C, D, G, H). AgomiR-223 treatment repressed expression of NLRP3, based on WB (Figures 6A, B) and IHC (Figures 6C, D) analysis in vivo. Consistent with in vitro results, increased miR-223 also reduced Keap1 protein in experimental mastitis, based on both WB (Figures 6E, F) and IHC (Figures 6G, H) of experimental mastitis. On the contrary, transient inhibition of murine miR-223 expression exacerbated mammary gland inflammation and oxidation (Figures 8A–H). Collectively, these data implicated miR-223 as modulating the NLRP3 inflammasome and Keap1-medidated oxidative stress pathway in experimental mastitis.

## Discussion

Our primary aim was to determine whether and how miR-223 regulated the NLRP3 inflammasome and Keap1-mediated oxidative stress, based on in vitro and in vivo models of LPS-induced inflammation. Our preliminary data were supportive of this hypothesis. It is well known that *E. coli* commonly causes acute and severe bovine mastitis [3, 36]. Furthermore, LPS, the main virulence factor of *E. coli*, is a major microbe-associated molecular pattern that evokes the bovine immune response [10] and oxidative stress [8] and induces the NLRP3 inflammasome and Keap1/Nrf2 pathway in various disease models [4, 6, 7, 16, 19, 37, 38]. Whole-mRNA transcriptome sequencing was used to explore differentially expressed genes (DEGs) that may be involved in the inflammatory signaling pathway and oxidative stress-related pathway in bovine mammary gland or bMECs stimulated by LPS [39, 40]. In the present study, based on integration of miRNA and mRNA expression data, miR-223, as a potential novel therapeutic target, has been strongly associated with bovine mammary disease [22, 25, 26, 41]. Although miR-223 mitigated inflammation and prevented collateral damage during infection and inflammation [14, 22, 25, 37, 38, 42], its function and regulation in response to NLRP3 or Keap1 treatment in bovine mastitis have not been well characterized.

NLRP3 is a unique NOD-like receptor family member that can be activated by inflammatory stimuli, including LPS, extracellular ATP, K + efflux, intracellular ROS, etc. [43, 44]. The NLRP3 inflammasome is activated in a two-step manner [13, 44]. In previous studies, LPS activated the NLRP3 inflammasome in epithelial cells [43, 45] and murine mammary tissue [46]. In our study, protein expression of NLRP3/caspase 1/IL-1β pathway was upregulated in bMECs with LPS and ATP, whereas treatment with MCC950 (NLRP3 inhibitor) blocked NLRP3 and its downstream proteins (Figures 2E, F). It was reported that LPS caused a TLR4-independent mitochondrial ROS (mtROS) accumulation and further NLRP3 inflammasome activation in other epithelial cells [43]. Cumulated free radicals induce cell damage, increasing the risk of developing mastitis [8]. In addition, the observed high-level of ROS induced by LPS in bMECs may also cause mitochondrial injury [47], which activate the NLRP3/caspase 1/IL-1β pathway, consistent with our model (Figures 2E, F, I, J). Furthermore, IL-1β, also an important proinflammatory factor, was reported to be triggered by ROS through activation of the NLRP3 inflammasome in epithelial cells [48].

NLRP3 inflammasome activity negatively controlled by miR-223 was first reported in 2012, with a report of an inverse correlation of miR-223 and NLRP3 expression in mononuclear cells of the myeloid lineage [49]. In the last decade, inverse associations between miR-223 and NLRP3 were reported in multiple tissues and cells, including colons of miR-223^−/y^ mice with colitis induced by dextran sulfate sodium (DSS) [50], lung macrophages [37], and livers of miR-223^−/−^ mice [38]. By combining results of dual-luciferase reporter assay (Figures 3A, B), we inferred that bta-miR-223 directly targeted *Bos taurus* NLRP3. Furthermore, bta-miR-223 mimics and inhibitor addition temporally controlled miR-223 release in bMECs; its steady and high expression suggested it could limit activation of the NLRP3 inflammasome (Figures 4A–D). In the present study, bta-miR-223 inhibited upregulation of caspase 1-p20 and IL-1β by downregulating NLRP3 in bMECs (Figures 4A, B). This underscored the importance of the association between dysregulated *Bos taurus* miRNAs and aberrantly modulating functional target genes in bMECs. In our in vivo experiment, injection of agomir (miR-223 activator) remarkably attenuated LPS-induced inflammation in the murine mammary gland, based on histopathology (Figures 5A, B). Furthermore, inhibition of miR-223 enhanced pathology of murine mastitis (Figures 7A, B) and increased expression of NLRP3 protein (Figures 8A–D), consistent with previous studies in cattle [14]. The NLRP3 inflammasome has a pivotal role in both chronic [38] and acute [37] inflammation; targeting inflammation at early disease stages is a therapeutic pillar to prevent disease progression. In brief, our results provided proof of concept that either transfection with synthetic miR-223 mimetics or direct inhibition of miR-223 has much potential to alter mammary inflammation.

Excessive oxidative stress is an important indicator of a pathological state. Several molecular mechanisms and signaling pathways can mediate oxidative stress [15, 17, 51]. In addition to resident and recruited immune cells in mammary gland, bMECs have been implicated in direct contact with environmental pathogens and in regulation of pro-inflammation and oxidative stress [2, 4, 6, 7, 9, 16, 18, 19, 51]. Firstly, we observed activation of Keap1/Nrf2/HO-1/SOD1 oxidative stress pathway in LPS-induced bMECs (Figures 2C, D), consistent with a previous report [6, 7]. Of note, the contrast in the result of H_2_O_2_ stimulation with bMECs (Additional files 2A, B) suggested that the cells were in a strong state of oxidative stress [51]. However, whether miRNA has a direct role in activating oxidative damage is poorly understood. In our in vitro experiments, miR-223 negatively regulated Keap1 expression through a conserved binding site within the 3’UTR region of *Bos taurus* Keap1 (Figures 3A, C), although we did not predict bta-miR-223 binding to the 3’UTR region of *Bos taurus* Nrf2. Moreover, treatment of bMECs with miR-223 mimicked downregulated expression of Keap1 and subsequently activated Nrf2 (Figures 4E, F). The Keap1/Nrf2 system acts as a key regulator for protecting against ROS, with a crucial role in antioxidant metabolism and response, as well as in prevention of mastitis [6–8]. Under unstressed conditions, both Nrf2 and Keap1 retained some expression in bMECs. Keap1 was identified as a critical repressor of Nrf2 activity and Keap1-Nrf2 complex is normally in a low activity state in the cytoplasm. However, under oxidative stress or other pathological stimuli, Nrf2 is dissociated from Keap1, and then translocates to the nucleus, and further activates a series of antioxidant enzymes [12]. Therefore, inhibition of Keap1 increases Nrf2 activity [17] under abundant miR-223 conditions, thereby controlling oxidative stress.

Notably, altered Keap1/Nrf2 axis enhanced activities of HO-1 and SOD1 in vitro, which eliminated a variety of active oxygen free radicals and activated antioxidant defenses [8, 12]. A recent study also reported elevated miR-223 as a negative regulator of Keap1 (both mRNA and protein levels) and upregulated levels of Nrf2, HO-1, SOD1 and SOD2 protein levels in type 2 diabetes mellitus (T2DM) induced human hepatocarcinoma cell line (HepG2) cell lines, thereby mitigating liver injury [30]. Therefore, our results emphasized miR-223 may be a novel Keap1-targeting miRNA and further activated the Nrf2 cascade in an experimental mastitis model.

In this study, LPS (50 μg/mL) decreased cell viability and increased LDH release in bMECs (Figures 1A, B), consistent with a previous report [9]. Thus, we inferred that cellular inflammation was related to the damaged cell membrane [52] and mitochondrial injury [47, 53]. Furthermore, following LPS stimulation concentrations of IL-1β, IL-18, TNF-α and IL-6 increased in bMECs supernatant after 12 h (Figures 1C–F), consistent with a characteristic rapid and acute inflammation [10]. Inflammatory responses have varied regulatory mechanisms, including NLRP3 inflammasome and TLR4/NF-κB pathway [5, 11, 14, 19]. Activation of such signaling pathways of inflammation has a valuable role in regulating the immune response.

Activation of signaling pathways of inflammation is meaningful in regulating immune responses, and characterizing proinflammatory and anti-inflammatory mechanisms is critical to prevent and treat disease. In this manuscript, we considered that contact and stimulation of the pathogen with bMECs were relevant for effector functions of immune defense in the udder [54, 55]. However, we are aware that systemic inflammation can affect oxidative and inflammatory stress responses to immune cells. Additionally, that mature miRNAs are likely involved in intercellular communication has been well documented, including exosomes [56], microvesicles [37] and lipoproteins [57]. miR-223 is one of the most abundant miRNAs in neutrophils [29], monocytes [57] and macrophages [37]. Expression of miR-223 was elevated in the murine mammary gland, whereas expression in bMECs was comparatively lower. Perhaps miR-223 acted as a signal transmitting molecule from myeloid cells to mammary epithelial cells and thus regulate inflammation, which should be a major focus for our future studies.

Taken together, we first identified that an LPS challenge increased inflammation and oxidative stress in bMECs. Then, our objective was to emphasize the role of miR-223 in regulation of the NLRP3 inflammasome and Keap1/Nrf2 oxidative stress pathway in mastitis models involving LPS treatment of bMECs and murine mammary glands.

**Figure 9:**
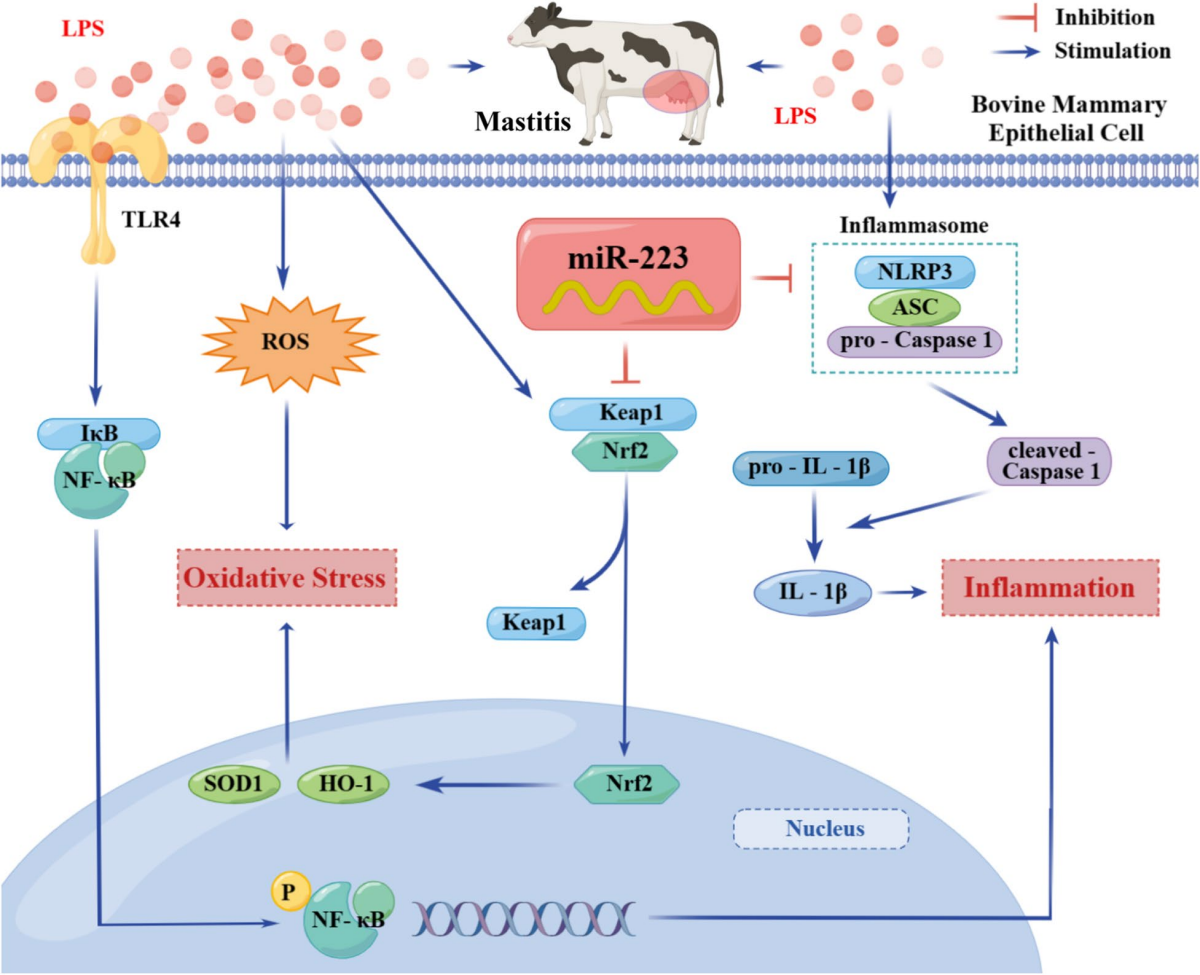
**A proposed model depicting the possible mechanism of bta-miR-223 on mediating inflammatory injuries and oxidative stress via regulation of NLRP3 inflammasome and Keap1/Nrf2 pathway**.

In summary, our study highlighted that targeting NLRP3 and Keap1 by therapeutic bta-miR-223 ameliorated the NLRP3 inflammasome and Keap1/Nrf2 signaling pathways to protect the mammary gland from inflammatory and oxidative injury (Figure 9). Importantly, bta-miR-223 has much promise as a novel target for treating bovine mastitis or other associated diseases.

### Supplementary Information

**Additional file 1. Establishment of bta-miR-223 mimics and bta-miR-223 inhibitor on immortalized bMECs.** Effects of 20 nmol/L or 50 nmol/L bta-miR-223 mimic **(A, B)**, 20 nmol/L or 50 nmol/L bta-miR-223 inhibitor **(A, B)** of protein expression of NLRP3 or Keap1. *p < 0.05 and **p < 0.01, differences compared to control group (mimics or inhibitor siRNA NC); &*p* < 0.05, differences between 20 nmol/L and 50 nmol/L bta-miR-223 mimics samples; aa*p* < 0.01, differences between 20 nmol/L and 50 nmol/L bta-miR-223 inhibitor samples.

**Additional file 2. Keap1/Nrf2 signaling pathway activation of bMECs induced by LPS and/or H**_**2**_**O**_**2**_** (500 μmol/L) for 3 h.** Protein expression of Keap1 and Nrf2 of bMECs induced by LPS and/or H_2_O_2_ (**A, B**). *p < 0.05 and **p < 0.01, differences compared to control group; ##*p* < 0.01, differences compared to LPS and H_2_O_2_-induced samples.

## Data Availability

All data generated or analyzed during this study are included in this published article.

## Abbreviations

miR-223: microRNA-223
NLRP3: nucleotide-binding oligomerization domain-like receptor containing pyrin domain 3
Keap1: Kelch like ECH-associated protein 1
Nrf2: nuclear factor erythroid 2-related factor 2
LPS: lipopolysaccharide
bMECs: bovine mammary epithelial cells
3’-UTR: 3'-Untranslated region
*E. coli*: *Escherichia coli*
ROS: reactive oxygen species
ASC: apoptosis-associated speck-like protein
miRNAs: microRNAs
DMEM: Dulbecco’s modified Eagle’s medium
ATP: Adenosine 5’-triphosphate
siRNA: short interfering RNA
WT: wild type
MUT: mutant type
NC: negative control
CCK-8: cell counting kit-8
LDH: Lactate dehydrogenase
ELISA: enzyme-linked immunosorbent assay
DCFH-DA: dichlorofluorescein diacetate
PBS: phosphate buffered solution
HE: hematoxylin and eosin
IHC: immunohistochemistry
HRP: horseradish peroxidase
DAB: 3,3’-Diaminobenzidine
FISH: fluorescence in situ hybridization
DAPI: 4,6-Diamidino-2-phenylindole
qRT-PCR: quantitative real-time PCR
WB: Western blotting
RIPA: radioimmunoprecipitation assay
BCA: bicinchoninic acid
NF-κB: nuclear factor-κ-gene binding
IκB: inhibitor of NF-κB
HO-1: heme oxygenase-1
SOD1: superoxide dismutase 1
TLR4: toll-like receptor 4
ECL: enhanced chemiluminescence
SD: standard deviation
DMSO: dimethyl sulfoxide
DEGs: differentially expressed genes
mtROS: mitochondrial reactive oxygen species
DSS: dextran sulfate sodium
T2DM: type 2 diabetes mellitus
HepG2: human hepatocarcinoma cell line
