# Supplementary figures and images for: MicroRNA miR-223 modulates NLRP3 and Keap1, mitigating lipopolysaccharide-induced inflammation and oxidative stress in bovine mammary epithelial cells and murine mammary glands
Source: Vet Res. 2023 Sep 14;54:78. doi: 10.1186/s13567-023-01206-5 (PMC10503159; doi:10.1186/s13567-023-01206-5)

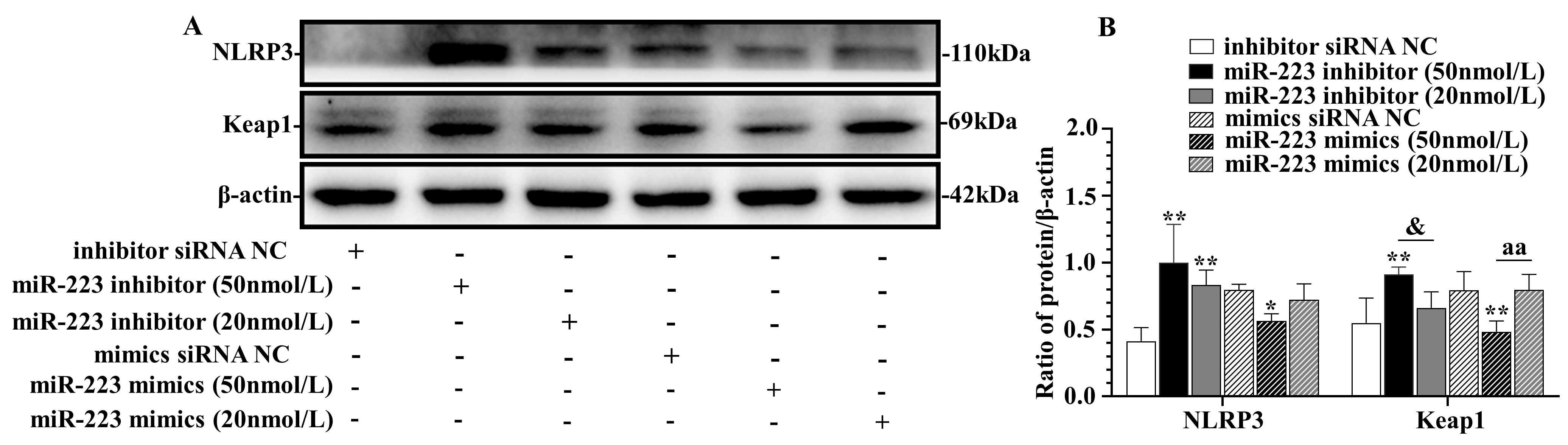

Supplement: Supplementary file 1 — Additional file 1. Establishment of bta-miR-223 mimics and bta-miR-223 inhibitor on immortalized bMECs. Effects of 20 nmol/L or 50 nmol/L bta-miR-223 mimic (A, B), 20 nmol/L or 50 nmol/L bta-miR-223 inhibitor (A, B) of protein expression of NLRP3 or Keap1. *p < 0.05 and **p < 0.01, differences compared to control group (mimics or inhibitor siRNA NC); &p < 0.05, differences between 20 nmol/L and 50 nmol/L bta-miR-223 mimics samples; aap < 0.01, differences between 20 nmol/L and 50 nmol/L bta-miR-223 inhibitor samples. [file 13567_2023_1206_MOESM1_ESM.tif]

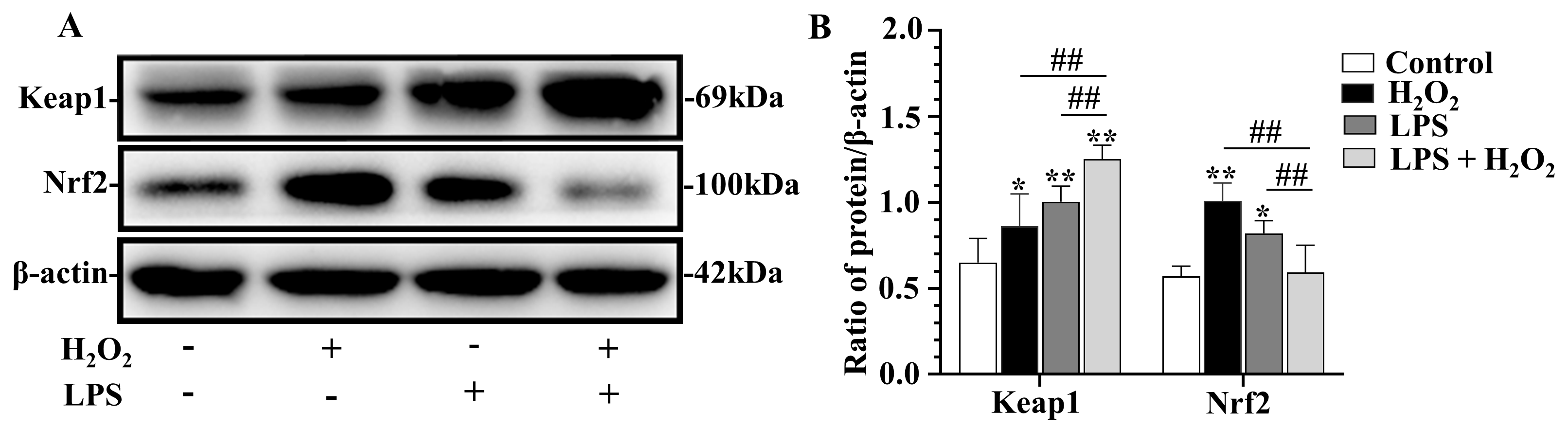

Supplement: Supplementary file 2 — Additional file 2. Keap1/Nrf2 signaling pathway activation of bMECs induced by LPS and/or H2O2 (500 μmol/L) for 3 h. Protein expression of Keap1 and Nrf2 of bMECs induced by LPS and/or H2O2 (A, B). *p < 0.05 and **p < 0.01, differences compared to control group; ##p < 0.01, differences compared to LPS and H2O2-induced samples. [file 13567_2023_1206_MOESM2_ESM.tif]
